# Genetic Mechanisms Driving Uterine Leiomyoma Pathobiology, Epidemiology, and Treatment

**DOI:** 10.3390/genes15050558

**Published:** 2024-04-27

**Authors:** Malini S. Ramaiyer, Eslam Saad, Irem Kurt, Mostafa A. Borahay

**Affiliations:** 1 Johns Hopkins University School of Medicine, Baltimore, MD 21205, USA; mramaiy1@jhmi.edu; 2 Department of Gynecology and Obstetrics, Johns Hopkins University, 720 Rutland Ave, Baltimore, MD 21205, USA; esaad2@jhmi.edu (E.S.); driremkurt@gmail.com (I.K.); 3 Faculty of Medicine, Selcuk University, 42000 Konya, Turkey

**Keywords:** uterine fibroids, cytogenetics, epidemiology, gene therapy

## Abstract

Uterine leiomyomas (ULs) are the most common benign tumor of the uterus. They can be associated with symptoms including abnormal uterine bleeding, pelvic pain, urinary frequency, and pregnancy complications. Despite the high prevalence of UL, its underlying pathophysiology mechanisms have historically been poorly understood. Several mechanisms of pathogenesis have been suggested, implicating various genes, growth factors, cytokines, chemokines, and microRNA aberrations. The purpose of this study is to summarize the current research on the relationship of genetics with UL. Specifically, we performed a literature review of published studies to identify how genetic aberrations drive pathophysiology, epidemiology, and therapeutic approaches of UL. With regards to pathophysiology, research has identified *MED12* mutations, *HMGA2* overexpression, fumarate hydratase deficiency, and cytogenetic abnormalities as contributors to the development of UL. Additionally, epigenetic modifications, such as histone acetylation and DNA methylation, have been identified as contributing to UL tumorigenesis. Specifically, UL stem cells have been found to contain a unique DNA methylation pattern compared to more differentiated UL cells, suggesting that DNA methylation has a role in tumorigenesis. On a population level, genome-wide association studies (GWASs) and epidemiologic analyses have identified 23 genetic loci associated with younger age at menarche and UL growth. Additionally, various GWASs have investigated genetic loci as potential drivers of racial disparities in UL incidence. For example, decreased expression of Cytohesin 4 in African Americans has been associated with increased UL risk. Recent studies have investigated various therapeutic options, including ten-eleven translocation proteins mediating DNA methylation, adenovirus vectors for drug delivery, and “suicide gene therapy” to induce apoptosis. Overall, improved understanding of the genetic and epigenetic drivers of UL on an individual and population level can propel the discovery of novel therapeutic options.

## 1. Introduction

Uterine leiomyoma (UL) has an estimated economic burden of USD 5.9 billion to USD 34.4 billion in the United States due to both direct costs of medical care and indirect costs [1]. This economic burden reflects the morbidity associated with UL—while 50% of individuals with UL are asymptomatic, UL symptoms include irregular bleeding, heavy menstrual bleeding, severe anemia, pelvic pressure and pain, infertility, and pregnancy complications [1,2]. Depending on the symptoms experienced, medical treatments include a levonorgestrel intrauterine device, gonadotropin-releasing hormone (GnRH) agonists, selective progesterone receptor modulators (SPRMs), and oral contraceptives [3,4]. Among these medical therapies, GnRH agonists have demonstrated the most efficacy in the reduction of UL volume and symptom alleviation [5]. However, these agents are recommended for a maximum use of 6 months, due to negative side effects including loss of bone density and diabetes [5]. As such, the main definitive and curative therapy for those experiencing symptoms remains hysterectomy [6]. This option is less than ideal for individuals who want to avoid surgery and/or desire to keep their uterus. Despite UL being the most common benign tumor of the female reproductive tract, medical treatment to address UL symptoms and associated complications has much room to grow.

**Figure 1:**
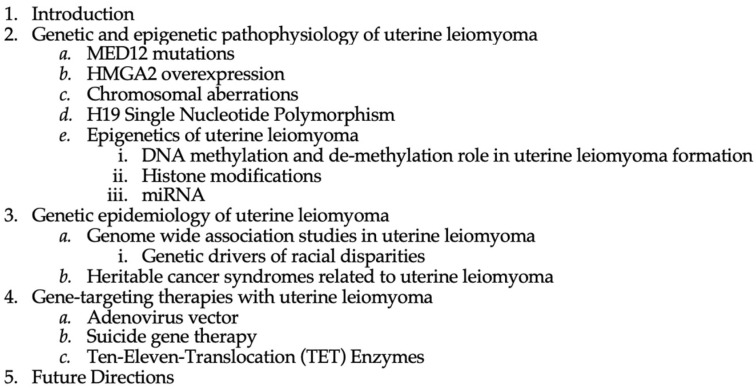
Review outline.

The main challenge in developing therapies for UL remains the unknown etiology of UL. While ULs are defined as benign tumors of the uterus, characterized as clonal and hormonally regulated, little is known about the pathogenesis leading to UL growth [7]. Consequently, current research is aimed at uncovering the genetic and epigenetic modifications driving the tumorigenesis of UL in various individuals [8]. Uncovering the genetic and epigenetic mechanisms behind UL is hypothesized to help develop personalized medical therapies for individuals diagnosed with UL [7]. This review aims to describe current research on the genetic and epigenetic mechanisms behind UL pathophysiology, UL genetic epidemiology, and current genetic therapies being developed for UL, in order to highlight areas of growth for UL therapeutics (Figure 1).

## 2. Genetic and Epigenetic Pathophysiology of Uterine Leiomyoma

**Figure 2:**
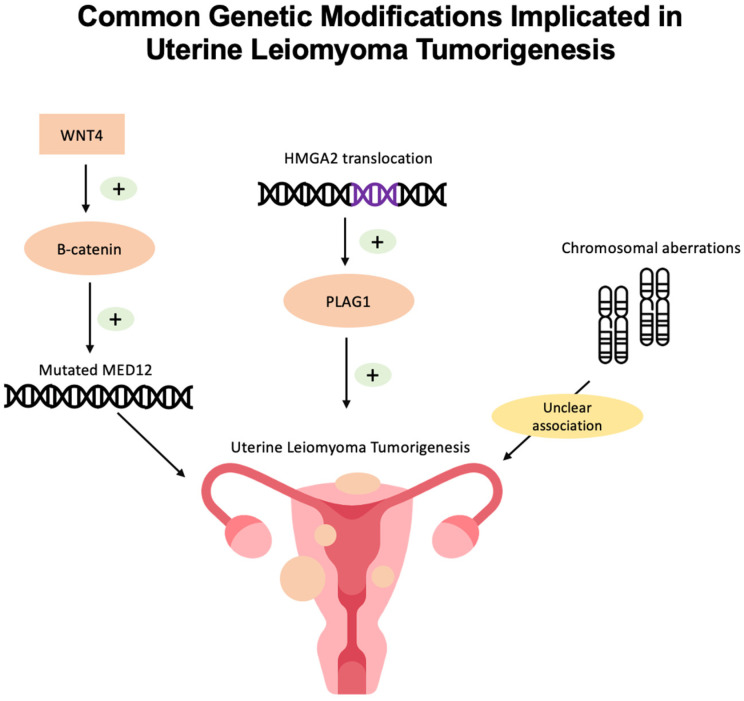
Common genetic modifications implicated in uterine leiomyoma tumorigenesis include MED12 mutations, HMGA2 translocation, and chromosomal aberrations.

Currently, the exact mechanism of UL tumorigenesis is unknown. However, genomic, genetic, and epigenetic studies have revealed a few common mutations in UL (Figure 2).

### 2.1. MED12 Mutations

The mediator complex subunit 12 (*MED12*) has been identified as the most common genetic mutation in UL [9]. The *MED12* complex is a 26-subunit transcriptional regulator that bridges DNA regulatory sequences to the RNA polymerase II initiation complex [10]. Makinen et al. examined 225 UL tumors from 80 patients and found that 70% of tumors contained alterations to MED12, specifically in exon 2 [10]. These mutations are predominantly deletion, insertion, and missense mutations [11]. However, the observed MED12 mutations were varied, as no individual mutation was repeated across tumors [11]. Moreover, Makinen et al. detected no correlation between *MED12* mutation status and patient age at hysterectomy [11]. Tumors lacking *MED12* mutations, however, were found to be larger [11]. Furthermore, Mi Je et al. evaluated whether MED12 mutations were specific to UL by evaluating 1862 tumor tissues, including a variety of carcinomas, leukemias, and stromal tumors. *MED12* mutations were only identified in 35 UL samples and 1 colon carcinoma sample. The specificity of *MED12* mutations to UL suggests that mutated *MED12* could be a therapeutic target in UL [12].

Additionally, *MED12* is implicated in the Wnt/β-catenin pathway, as β-catenin binds *MED12* to activate transcription. Furthermore, mutated *MED12* is hypothesized to increase Wnt/β-catenin signaling. Markowski et al. found that UL with *MED12* mutations expressed significantly higher levels of the gene encoding WNT4, compared with UL without *MED12* mutations [13]. Activated β-catenin has demonstrated UL-like growth in mouse models, and future research can target the link between *MED12* mutations and the Wnt/β-catenin pathway to develop novel treatment options [13]. More recently, CRISPR-Cas9 has been utilized to engineer mutant *MED12* UL cells and develop reliable UL cellular models, establishing a novel platform to further characterize UL with *MED12* mutations and develop UL therapeutic targets [14].

### 2.2. HMGA2 Overexpression

The high mobility group AT-hook 2 (*HMAG2*) gene encodes a protein which regulates transcription [15]. Overexpression of *HMAG2* has been observed to be the second most common genetic driver of UL following *MED12* [16]. Mehine et al. state that HMAG2 and *MED12* are the two most common genes contributing to the growth of up to 80–90% of all UL [17]. Galindo et al. analyzed 20 UL tumors and found overexpression of *HMGA2* mRNA measured by quantitative PCR in UL compared to myometrial tissues [16]. The overexpression of *HMAG2* is hypothesized to be due to chromosomal translocations [18].

Conflicting data exist on the relationship between *HMAG2* and *MED12*. While one study by Bertsch et al. demonstrated that *HMGA2* overexpression was found only in UL with no MED12 mutation [19], Galindo et al. found that *HMGA2* overexpression and MED12 mutations frequently co-exist [16]. Further research is warranted in this area to understand whether *HMAG2* and *MED12* contribute to tumorigenesis independently and in tandem.

Additionally, Mehine et al. investigated 94 UL tissue samples and found that UL with *HMGA2* aberrations also had upregulated proto-oncogene pleomorphic adenoma gene 1 (PLAG1). Given these results, Mehine et al. propose that *HMGA2* may play a role in PLAG1 activation, ultimately contributing to tumorigenesis [17].

### 2.3. Chromosomal Aberrations

Chromosomal abnormalities are observed in about 40–50% of ULs [16]. The characteristic translocation associated with UL is t(12; 14) (q15; q23~q24), seen in 20% of karyotypically abnormal ULs [8]. Chromosomal rearrangements in UL are found to target two human *HMGC* gene loci [13]. Specifically, region 12q13~q15 contained the *HMGA2* gene in UL samples [20]. Overall, there is a heterogeneity among chromosomal aberrations found in UL, which reflects the pathologic and clinical heterogeneity in UL [20]. The relevance of these chromosomal changes to UL tumorigenesis has not yet been established; however, these chromosomal aberrations serve as molecular guides to genetic aberrations causing UL development [20].

### 2.4. H19 Single-Nucleotide Polymorphism

H19 long non-coding RNA (lncRNA) has been implicated in several fibrotic states of the liver, lung, and kidney [21,22,23] and is overexpressed in placental and fetal tissue [24]. In their 2019 study, Cao et al. investigate H19 lncRNA and demonstrate that H19 lncRNA regulates the expression of UL driver genes, including *MED12*, *HMGA2*, and *TET3* [24]. As such, Cao et al. show that H19 lncRNA promotes UL tumorigenesis and hypothesize that the SNP serves as a “master regulator” of UL driver genes [24].

### 2.5. Epigenetics of Uterine Leiomyoma

The term “epigenetics” describes phenotypic modifications brought about by changed gene expression that are not caused by variations in the DNA sequence. There are three major mechanisms of epigenetic regulation: (a) DNA methylation mediated by DNA methyltransferases as well as active and passive DNA demethylation, (b) modification of histone proteins, and (c) microRNAs [25].

#### 2.5.1. DNA Methylation and Demethylation Role in Uterine Leiomyoma Formation

Aberrant DNA methylation, linked to aberrant gene expression, is one of the distinguishing characteristics of tumors. Sato et al. found that there are ten UL genes—*ALX1*, *CBLN1*, *CORIN*, *DUSP6*, *FOXP1*, *GATA2*, *IGLON5*, *NPTX2*, *NTRK2*, and *STEAP4*—that are hypermethylated, while two genes—*PART1* and *PRL*—are hypomethylated [26]. Furthermore, there is evidence suggesting that DNA methylation may play a role in UL stem cell regulation [27]. Liu et al. investigated the progression of UL cells: (1) UL stem cell-like cells (LSCs), (2) UL intermediate cells (LICs), and (3) differentiated UL cells (LDCs) [27]. Liu et al. determined that LSCs contained a “unique methylome” compared to LICs and LDCs, suggesting that DNA methylation may contribute to the initial differentiation of LSCs [26]. These findings highlight the need for further investigation into the differentiation processes of LSCs as they relate to DNA methylation, in order to identify a point of intervention in inhibiting UL tumorigenesis [27].

Several genes have been implicated in UL tumorigenesis through investigation of DNA methylated or demethylated loci. Demethylation leads to increased expression of genes, whereas methylation leads to decreased expression of genes. Notably, demethylation of the HMGA2 gene was identified, which explains findings of upregulated HMGA2 within UL [28]. Furthermore, Carbajo-García et al. identified oncogenes (*PRL*, *ATP8B4*, *CEMIP*, *ZPMS2-AS1*, *RIMS2*, and *TFAP2C*) which were demethylated and consequently upregulated [28]. Conversely, Carbajo-Garcia et al. also identified hypermethylation and consequent downregulation of tumor suppressor genes (*EFEMP1*, *FBLN2*, *ARHGAP10*, and *HTATIP2*) within UL [29].

Examining the epigenetics of UL within the context of racial disparities in incidence, Paul et al. examined the DNA methylation and transcriptome of UL [30]. Stress-related changes promoting UL tumorigenesis are proposed to occur through altered DNA methylation [30]. Paul et al. clustered myometrial samples and UL samples using RNA-Seq and found that RNA-Seq myometrial cluster 1 had a statistically higher proportion among Black individuals whereas RNA-Seq myometrial cluster 2 had a statistically higher proportion among White individuals. No significant difference was found in UL sample RNA-Seq clusters based on race [30]. These findings suggest that the molecular basis of fibroid tissue itself does not vary; however, differences on a molecular level in the myometrium prior to UL development may predispose individuals to UL tumorigenesis. Paul et al. hypothesize the difference in myometrial RNA-Seq clusters in myometrium could be explained by shared experiences or exposures among specific races contributing to differential gene expression, reflecting a potential epigenetic modification that occurs through lived experiences [30].

#### 2.5.2. Histone Modifications

Histone alterations play a crucial role in chromatin packaging and gene expression control. The stability of the genome may be impacted by changes in histone modifications, which may also interfere with gene expression patterns and cause a variety of disorders, including cancer [31]. Acetylated Lysine 27 of histone 3 (H3K27ac) has been implicated in several tumors, including gastric, lung, and ovarian [32]. Carbajo-Garcia et al. investigated histone acetylation in UL by investigating whether H3K27ac is implicated in UL pathophysiology [32]. They used the histone deacetylases (HDACs) inhibitor suberoylanilide hydroxamic acid (SAHA). Utilizing RNA-seq and CHIP-seq for H3K27ac in UL vs. myometrial tissue, Carbajo-Garcia et al. found H3K27ac levels were lower in UL than in MM [31]. These findings, subsequently validated by qRT-PCR of SAHA-treated UL cells, suggest that histone acetylation promotes tumor suppression in UL cells and highlight that targeting histone modification is a potential therapeutic approach for reducing UL growth [33].

#### 2.5.3. miRNA

Gene expression is regulated by endogenous microRNAs (miRNAs), which are tiny non-coding RNAs. Specific circumstances may cause miRNAs to act as tumor suppressors or oncogenes. A number of cancer hallmarks have been demonstrated to be impacted by dysregulated miRNAs, including the ability to maintain proliferative signals, elude growth suppressors, withstand cell death, initiate invasion and metastasis, and stimulate angiogenesis [34]. In the pathogenesis of UL, miRNAs have been implicated as epigenetic mediators, promoting UL development via altered expression of proliferative, apoptotic, angiogenic, and ECM-forming genes [35]. In fact, expression of 46 miRNA species has been found to vary between normal myometrium and UL [36]. In UL, 19 miRNA species were found to be overexpressed and 27 miRNA species under expressed [36]. Real-time reverse transcriptase PCR was used to corroborate these results for a subset of miRNAs (miRNAs 21, 34a, 125b, 139, and 323) [36].

Focusing on miRNA-21, as a known mediator of tumor suppressor genes, Cardozo et al. specifically investigated the functional significance of miRNA-21. In cancer biology, broadly, miRNA-21 has been implicated and found to inhibit tumor suppressors, increase cell proliferation, and promote tumorigenesis [35]. Considered a “profibrogenic”, mi-RNA-21 is upregulated in UL and myometrial cells; however, Cardozo et al. found that miRNA-21 overexpression caused UL cells to proliferate more rapidly than myometrial cells [35].

Conversely, Huang et al. and found that high expression of miRNA-29 could inhibit UL cell growth through inhibition of the STAT3 signaling pathway [37]. As such, Marsh et al. showed that the miRNA-29 family is consistently downregulated in UL tissue, compared to myometrial tissue [38] This downregulation is hypothesized to play a role in the higher collagen content in UL as compared to the myometrium [38]. These are just two examples of the several miRNAs being investigated in the context of UL tumorigenesis. Further research should investigate how to utilize various miRNAs in the treatment of UL.

## 3. Genetic Epidemiology of Uterine Leiomyoma

As highlighted in the previous section, genetic alterations are thought to be one of the major contributors to the development of uterine leiomyomas by promoting the transformation of healthy myometrial stem cells into tumor-initiating ones [39]. Building upon this research, recent studies have investigated the genetics of UL on a population level, specifically describing heritable genetic syndromes and utilizing GWASs to identify common loci implicated in UL.

### 3.1. Genome-Wide Association Studies in Uterine Leiomyoma

Genome-wide association search (GWAS) and genome-wide single-nucleotide polymorphism analysis studies have detected variants in over 50 genes associated with predisposition to UL, including *p53*, telomerase reverse transcriptase (*TERT*), telomerase RNA component (*TERC*), and ATM serine/threonine kinase (*ATM*) [40]. Additionally, hormone-associated genes, also observed in endometriosis and breast cancer, have been associated with UL, including cell division cycle 42 (*CDC42/WNT4*), *GREB1*, minichromosome maintenance 8 homologous recombination repair factor (*MCM8*), and spectrin repeat containing nuclear envelope protein 1 (*SYNE1/ESR1*) [41].

One of the largest GWASs conducted in the Japanese population revealed different associations between gynecological diseases. The gamma-aminobutyric acid type B receptor subunit 2 (*GABBR2*) locus was discovered to pertain to combined phenotypes of UL and ovarian cancer, while the SH3 domain containing the GRB2-like 3/basonuclin zinc finger protein 1 (*SH3GL3/BNC1*) locus was associated solely with UL [42]. Likewise, a novel locus near the *LINC00485* gene, associated with UL in the Japanese population, was reported [43].

Younger age at menarche has historically been recognized as a risk factor for UL tumorigenesis [44]. As such, age at menarche has been investigated on a genomic level. Ponoarenko et al. identified 23 genetic loci associated with UL, of which 16 showed an association with either age at menarche or BMI [45]. In another GWAS conducted on individuals of Han Chinese descent, a variant in STE20-like kinase (*SLK*) increased the risk of UL development via a mechanism independent of age at menarche, while another variant in HLA class II histocompatibility antigen, DO beta chain (*HLA-DOB*) decreased the risk through its association with age at menarche [46].

GWASs have also demonstrated a genetic overlap between UL and breast cancer, particularly in the ER+ subtype [47], as well as with endometriosis, involving genes such as *WNT4/CDC42*, *GREB1*, *ESR1*, and follicle-stimulating hormone subunit beta (*FSHB*) [48,49]. Additionally, there is genetic overlap between UL and endometrial cancer, with implicated genes including CLPTM1-like (*CLPTM1L*), microRNA 4457 (*MIR4457*), TERT, WT1 transcription factor (*WT1*), and WT1 antisense RNA (*WT1-AS*) [50].

#### 3.1.1. Genetic Drivers of Racial Disparities

Racial disparities in UL incidence, burden of disease, and age of diagnosis have been established on an epidemiologic level. With a higher incidence of UL compared to White individuals, Black individuals are additionally diagnosed at younger ages, experience longer symptom duration, and demonstrate larger UL volume [51]. Therefore, in order to provide more individualized treatment options, research efforts have focused on genetic drivers of this racial disparity.

In 2017, Hellwege et al. conducted a multi-stage GWAS in African American individuals. Utilizing UL and control myometrium samples, the study evaluated for genetic associations to identify risk loci for UL among African American individuals [52]. Hellwege et al. found that decreased expression of Cytohesin 4 (CYTH4), which is involved in gene expression in the thyroid [46], was significantly associated with UL risk.

Later, in 2019, Edwards et al. expanded upon this work by investigating individuals of African and European ancestry in the UK with and without UL. This analysis identified a variant in *CDC42/WNT4* is less likely to occur in individuals of African descent (OR = 0.84) compared to European descent (OR = 1.16) [53]. Following these novel initial findings, further research is necessary to understand the biological relevance of specific variants to UL tumorigenesis and how they may contribute to increasing UL incidence in Black individuals.

#### 3.1.2. Heritable Syndromes Related to Uterine Leiomyoma

Heterozygous germline fumarate hydratase (*FH*) mutations have been demonstrated to be associated with an autosomal dominant disease named multiple cutaneous and uterine leiomyomatosis (MCUL) [54], as well as a rare disorder known as hereditary leiomyomatosis and renal cell carcinoma (HLRCC) [55,56]. HLRCC presents with increased cutaneous and uterine leiomyoma, caused by heterozygous pathogenic germline variants in the *FH* gene [56]. Variant fumarate hydratase leads to fumarate accumulation in UL cells, leading to formation of S-(2-succino)-cysteine [57]. The formation of these cysteine residues, as such, indicates *FH* aberration and detection of S-(2-succino)-cysteine-positive UL can be used to identify *FH*-deficient UL in research and clinically [57]. Identification of *FH* deficiency may be a critical step in the diagnostic workup of patients suspected to have HLRCC [57]. While numerous studies have outlined a plausible role of *FH* in syndromic uterine leiomyomas [58], its involvement in non-syndromic uterine leiomyomas remains obscure [54,55,59].

## 4. Gene-Targeting Therapies in Uterine Leiomyoma

**Figure 3:**
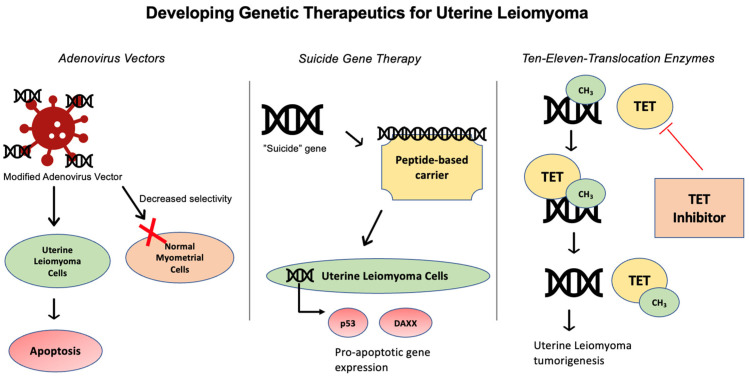
Targeted gene therapies for uterine leiomyoma include use of adenovirus vectors, suicide gene therapy, and ten-eleven translocation enzyme inhibitors.

Several studies have investigated UL gene therapy through different approaches, in an attempt to develop non-surgical, non-hormonal treatment options [60] (Figure 1). UL can be amenable for targeted delivery via local injection of gene-based vectors, due to their localized nature [61] (Figure 3).

### 4.1. Adenovirus Vector

Adenoviral vectors have been investigated as a drug-delivery platform for targeting human leiomyoma (HuLM) cells [61]. Specifically, the adenovirus serotype 5 historically used for gene therapies in other tumor pathologies has been observed to bind to coxsackie-adenovirus receptor (CAR) [61]. With regards to UL, however, CAR has been found to be downregulated in UL, compared to myometrial tissue [61]. Therefore, Hassan et al. tested several modified adenovirus vectors which utilize CAR-independent pathways to target UL tumor cells and found several modified adenovirus vectors with increased selectivity toward HuLM cells compared to immortalized human myometrial cells [62]. Abdelaziz et al. built upon this work to develop adenovirus vector Ad-SSTR-RGD-TK followed by Ganciclovir (GCV) and found this therapy to be effective in selectively inducing apoptosis in in vitro and in vivo studies. Future directions include testing this therapy in animal models, before moving to clinical trials [61].

### 4.2. Suicide Gene Therapy

Suicide gene therapy (SGT) is another modality being developed to improve drug delivery to UL tissue [63], in which a “suicide” gene is introduced to tumors. SGT can be introduced to tumor cells via viral or bacterial vectors and subsequently converts a pro-drug into a pro-apoptotic compound within the targeted cell [64]. SGT has demonstrated promising results in vitro and in vivo for a wide variety of cancers, yet application to human studies has not yet reached fruition [64]. With respect to UL, the herpes simplex virus-thymidine kinase/Ganciclovir (HSV-TK/GCV) system is regarded as an efficacious SGT system and has been tested in various gene therapy studies targeting UL [63]. Hassan et al. utilized adenovirus vectors (Ad) to deliver HSV-TK/GCV in Eker rat models with MRI-confirmed UL and found that Ad-HSV-TK/GCV treatment significantly reduced UL volume [65]. These findings established promising pre-clinical results to develop an Ad-HSV-TK/GCV system for human UL therapy.

SGT has been investigated with drug delivery platforms beyond adenovirus vector models. Specifically, peptide-based carriers have been found to have several favorable traits for drug delivery [63]. Unlike viral vectors, peptide-based carriers have the advantage of not triggering immune responses, carrying a higher capacity to transport nucleic acids, and having potential for large-scale production [63]. Most recently, Egorava et al. employed ternary DNA polyplexes to deliver HSV-TK into primary UL cells and detected an increase in apoptosis gene expression [63]. The DNA polyplexes are defined as cRGD-ligand-decorated-polyanion-coated ternary polyplexes, in which the polyanion coating achieves serum resistance [63]. After employing ternary DNA polyplex SGT, Egorava et al. found expression of the *HSV-TK* gene in UL tissue led to an increase in pro-apoptotic *p53* and *DAXX* genes, demonstrating the potential of using ternary DNA polyplexes for SGT [63].

### 4.3. Ten-Eleven Translocation (TET) Enzymes

Beyond genetic therapies, therapies targeting the epigenetics of UL are also being developed. Epigenetic mechanisms in UL involve DNA methylation and demethylation to regulate gene expression. These reactions are thought to be mediated by ten-eleven translocation proteins (TETs)—DNA demethylation mediated by TET has been proposed as leading to UL formation [66]. Broadly, TETs have been implicated in several cancers, including myelodysplastic syndromes and myeloproliferative tumors [67]. With regards to UL, it is hypothesized that improved understanding of TET-mediated epigenetic imbalance could generate new therapeutic targets for UL [66]. The advantage of epigenetic therapies is that epigenetic modifications are reversible and avoid the potential ethical complications associated with genetic changes induced by medical therapy [66]. Specific TET inhibitor agents have not yet been developed in the context of UL.

## 5. Future Directions

UL is the most common benign tumor of the female reproductive tract, and targeted gene therapy for UL remains a growing area of research. As highlighted in this review, several genetic and epigenetic alterations have been identified in association with UL tumorigenesis. For identified genetic mutations, including MED12, HMGA2, and chromosomal translocations, UL therapeutics may target these loci in order to modulate UL growth. Similarly, improved understanding of epigenetic modifications, such as DNA demethylation and histone acetylation, can lead to the development of targeted therapies. Specifically, TET enzymes, adenovirus vectors, and suicide gene therapy are the agents with the most promising potential as medical therapies for UL. As many of these therapies have currently achieved success in in vivo pre-clinical models, future steps include investigating the use of these therapies in a clinical setting.

## References

[1] Marsh E.E., Al-Hendy A., Kappus D., Galitsky A., Stewart E.A., Kerolous M. (2018). Burden, Prevalence, and Treatment of Uterine Fibroids: A Survey of U.S. Women. J. Women’s Health.

[2] Gupta S., Jose J., Manyonda I. (2008). Clinical presentation of fibroids. Best. Pract. Res. Clin. Obstet. Gynaecol..

[3] De La Cruz M.S.D., Buchanan E.M. (2017). Uterine Fibroids: Diagnosis and Treatment. Am. Fam. Physician.

[4] Vilos G.A., Allaire C., Laberge P.-Y., Leyland N., Vilos A.G., Murji A., Chen I. (2015). The management of uterine leiomyomas. J. Obstet. Gynaecol. Can..

[5] (2012). Gonadotropin Releasing Hormone (GnRH) Analogues. LiverTox: Clinical and Research Information on Drug-Induced Liver Injury. National Institute of Diabetes and Digestive and Kidney Diseases.

[6] (2021). Management of Symptomatic Uterine Leiomyomas: ACOG Practice Bulletin, Number 228. Obstet. Gynecol..

[7] Commandeur A.E., Styer A.K., Teixeira J.M. (2015). Epidemiological and genetic clues for molecular mechanisms involved in uterine leiomyoma development and growth. Hum. Reprod. Update.

[8] Medikare V., Kandukuri L.R., Ananthapur V., Deenadayal M., Nallari P. (2011). The Genetic Bases of Uterine Fibroids; A Review. J. Reprod. Infertil..

[9] Ciavattini A., Di Giuseppe J., Stortoni P., Montik N., Giannubilo S.R., Litta P., Islam S., Tranquilli A.L., Reis F.M., Ciarmela P. (2013). Uterine Fibroids: Pathogenesis and Interactions with Endometrium and Endomyometrial Junction. Obstet. Gynecol. Int..

[10] Mäkinen N., Mehine M., Tolvanen J., Kaasinen E., Li Y., Lehtonen H.J., Gentile M., Yan J., Enge M., Taipale M. (2011). MED12, the mediator complex subunit 12 gene, is mutated at high frequency in uterine leiomyomas. Science.

[11] Sabeh M.E., Saha S.K., Afrin S., Islam M.S., Borahay M.A. (2021). Wnt/β-catenin Signalling Pathway in Uterine Leiomyoma: Role in Tumor Biology and Targeting Opportunities. Mol. Cell Biochem..

[12] Je E.M., Kim M.R., Min K.O., Yoo N.J., Lee S.H. (2012). Mutational analysis of MED12 exon 2 in uterine leiomyoma and other common tumors. Int. J. Cancer..

[13] Markowski D.N., Bartnitzke S., Löning T., Drieschner N., Helmke B.M., Bullerdiek J. (2012). MED12 mutations in uterine fibroids--their relationship to cytogenetic subgroups. Int. J. Cancer..

[14] Buyukcelebi K., Chen X., Abdula F., Duval A., Ozturk H., Seker-Polat F., Jin Q., Yin P., Feng Y., Wei J.-J. (2023). Engineered MED12 mutations drive uterine fibroid-like transcriptional and metabolic programs by altering the 3D genome compartmentalization. Res Sq..

[15] Baranov V.S., Osinovskaya N.S., Yarmolinskaya M.I. (2019). Pathogenomics of Uterine Fibroids Development. Int. J. Mol. Sci..

[16] Galindo L.J., Hernández-Beeftink T., Salas A., Jung Y., Reyes R., de Oca F.M., Hernández M., Almeida T.A. (2018). HMGA2 and MED12 alterations frequently co-occur in uterine leiomyomas. Gynecol. Oncol..

[17] Mehine M., Kaasinen E., Heinonen H.-R., Mäkinen N., Kämpjärvi K., Sarvilinna N., Aavikko M., Vähärautio A., Pasanen A., Bützow R. (2016). Integrated data analysis reveals uterine leiomyoma subtypes with distinct driver pathways and biomarkers. Proc. Natl. Acad. Sci. USA.

[18] Mäkinen N., Kämpjärvi K., Frizzell N., Bützow R., Vahteristo P. (2017). Characterization of MED12, HMGA2, and FH alterations reveals molecular variability in uterine smooth muscle tumors. Mol. Cancer.

[19] Bertsch E., Qiang W., Zhang Q., Espona-Fiedler M., Druschitz S., Liu Y., Mittal K., Kong B., Kurita T., Wei J.-J. (2014). MED12 and HMGA2 mutations: Two independent genetic events in uterine leiomyoma and leiomyosarcoma. Mod. Pathol..

[20] Sandberg A.A. (2005). Updates on the cytogenetics and molecular genetics of bone and soft tissue tumors: Leiomyoma. Cancer Genet. Cytogenet..

[21] Xie H., Xue J.D., Chao F., Jin Y.F., Fu Q. (2016). Long non-coding RNA-H19 antagonism protects against renal fibrosis. Oncotarget.

[22] Song Y., Liu C., Liu X., Trottier J., Beaudoin M., Zhang L., Pope C., Peng G., Barbier O., Zhong X. (2017). H19 promotes cholestatic liver fibrosis by preventing ZEB1-mediated inhibition of epithelial cell adhesion molecule. Hepatology.

[23] Lu Q., Guo Z., Xie W., Jin W., Zhu D., Chen S., Ren T. (2018). The lncRNA H19 Mediates Pulmonary Fibrosis by Regulating the miR-196a/COL1A1 Axis. Inflammation.

[24] Cao T., Jiang Y., Wang Z., Zhang N., Al-Hendy A., Mamillapalli R., Kallen A.N., Kodaman P., Taylor H.S., Li D. (2019). H19 lncRNA identified as a master regulator of genes that drive uterine leiomyomas. Oncogene.

[25] Yang Q., Mas A., Diamond M.P., Al-Hendy A. (2016). The Mechanism and Function of Epigenetics in Uterine Leiomyoma Development. Reprod. Sci..

[26] Sato S., Maekawa R., Yamagata Y., Tamura I., Lee L., Okada M., Jozaki K., Asada H., Tamura H., Sugino N. (2016). Identification of uterine leiomyoma-specific marker genes based on DNA methylation and their clinical application. Sci. Rep..

[27] Liu S., Yin P., Xu J., Dotts A.J., Kujawa S.A., Coon V J.S., Zhao H., Shilatifard A., Dai Y., Bulun S.E. (2020). Targeting DNA Methylation Depletes Uterine Leiomyoma Stem Cell–enriched Population by Stimulating Their Differentiation. Endocrinology.

[28] George J.W., Fan H., Johnson B., Carpenter T.J., Foy K.K., Chatterjee A., Patterson A.L., Koeman J., Adams M., Madaj Z.B. (2019). Integrated Epigenome, Exome, and Transcriptome Analyses Reveal Molecular Subtypes and Homeotic Transformation in Uterine Fibroids. Cell Rep..

[29] Carbajo-García M.C., Corachán A., Juárez-Barber E., Monleón J., Payá V., Trelis A., Quiñonero A., Pellicer A., Ferrero H. (2022). Integrative analysis of the DNA methylome and transcriptome in uterine leiomyoma shows altered regulation of genes involved in metabolism, proliferation, extracellular matrix, and vesicles. J. Pathol..

[30] Paul E.N., Grey J.A., Carpenter T.J., Madaj Z.B., Lau K.H., Givan S.A., Burns G.W., Chandler R.L., Wegienka G.R., Shen H. (2022). Transcriptome and DNA methylome analyses reveal underlying mechanisms for the racial disparity in uterine fibroids. JCI Insight..

[31] Audia J.E., Campbell R.M. (2016). Histone Modifications and Cancer. Cold Spring Harb. Perspect. Biol..

[32] Carbajo-García M.C., de Miguel-Gómez L., Juárez-Barber E., Trelis A., Monleón J., Pellicer A., Flanagan J.M., Ferrero H. (2022). Deciphering the Role of Histone Modifications in Uterine Leiomyoma: Acetylation of H3K27 Regulates the Expression of Genes Involved in Proliferation, Cell Signaling, Cell Transport, Angiogenesis and Extracellular Matrix Formation. Biomedicines.

[33] Carbajo-García M.C., Juarez-Barber E., Segura-Benítez M., Faus A., Trelis A., Monleón J., Carmona-Antoñanzas G., Pellicer A., Flanagan J.M., Ferrero H. (2023). H3K4me3 mediates uterine leiomyoma pathogenesis via neuronal processes, synapsis components, proliferation, and Wnt/β-catenin and TGF-β pathways. Reprod. Biol. Endocrinol..

[34] Ali Syeda Z., Langden S.S.S., Munkhzul C., Lee M., Song S.J. (2020). Regulatory Mechanism of MicroRNA Expression in Cancer. Int. J. Mol. Sci..

[35] Cardozo E.R., Foster R., Karmon A.E., Lee A.E., Gatune L.W., Rueda B.R., Styer A.K. (2018). MicroRNA 21a-5p overexpression impacts mediators of extracellular matrix formation in uterine leiomyoma. Reprod. Biol. Endocrinol..

[36] Marsh E.E., Lin Z., Yin P., Milad M., Chakravarti D., Bulun S.E. (2008). Differential expression of microRNA species in human uterine leiomyoma versus normal myometrium. Fertil. Steril..

[37] Huang D., Xue H., Shao W., Wang X., Liao H., Ye Y. (2022). Inhibiting effect of miR-29 on proliferation and migration of uterine leiomyoma via the STAT3 signaling pathway. Aging.

[38] Marsh E.E., Steinberg M.L., Parker J.B., Wu J., Chakravarti D., Bulun S.E. (2016). Decreased expression of microRNA-29 family in leiomyoma contributes to increased major fibrillar collagen production. Fertil. Steril..

[39] Yang Q., Ciebiera M., Bariani M.V., Ali M., Elkafas H., Boyer T.G., Al-Hendy A. (2022). Comprehensive Review of Uterine Fibroids: Developmental Origin, Pathogenesis, and Treatment. Endocr. Rev..

[40] Välimäki N., Kuisma H., Pasanen A., Heikinheimo O., Sjöberg J., Bützow R., Sarvilinna N., Heinonen H.-R., Tolvanen J., Bramante S. (2018). Genetic predisposition to uterine leiomyoma is determined by loci for genitourinary development and genome stability. eLife.

[41] Rafnar T., Gunnarsson B., Stefansson O.A., Sulem P., Ingason A., Frigge M.L., Stefansdottir L., Sigurdsson J.K., Tragante V., Steinthorsdottir V. (2018). Variants associating with uterine leiomyoma highlight genetic background shared by various cancers and hormone-related traits. Nat. Commun..

[42] Masuda T., Low S.-K., Akiyama M., Hirata M., Ueda Y., Matsuda K., Kimura T., Murakami Y., Kubo M., Kamatani Y. (2020). GWAS of five gynecologic diseases and cross-trait analysis in Japanese. Eur. J. Hum. Genet..

[43] Sakai K., Tanikawa C., Hirasawa A., Chiyoda T., Yamagami W., Kataoka F., Susumu N., Terao C., Kamatani Y., Takahashi A. (2020). Identification of a novel uterine leiomyoma GWAS locus in a Japanese population. Sci. Rep..

[44] Qu Y., Chen L., Guo S., Liu Y., Wu H. (2023). Genetic Liability to Multiple Factors and Uterine Leiomyoma Risk: A Mendelian Randomization Study. Front. Endocrinol..

[45] Ponomarenko I., Reshetnikov E., Polonikov A., Verzilina I., Sorokina I., Yermachenko A., Dvornyk V., Churnosov M. (2020). Candidate Genes for Age at Menarche Are Associated with Uterine Leiomyoma. Front. Genet..

[46] Tai A.S., Lin R.T., Lin Y.C., Wang C.H., Lin S.H., Imoto S. (2022). Genome-wide causal mediation analysis identifies genetic loci associated with uterine fibroids mediated by age at menarche. Hum. Reprod..

[47] Wu X., Xiao C., Han Z., Zhang L., Zhao X., Hao Y., Xiao J., Gallagher C.S., Kraft P., Morton C.C. (2022). Investigating the shared genetic architecture of uterine leiomyoma and breast cancer: A genome-wide cross-trait analysis. Am. J. Hum. Genet..

[48] McGrath I.M., Montgomery G.W., Mortlock S. (2023). Insights from Mendelian randomization and genetic correlation analyses into the relationship between endometriosis and its comorbidities. Hum. Reprod. Update.

[49] Gallagher C.S., Mäkinen N., Harris H.R., Rahmioglu N., Uimari O., Cook J.P., Shigesi N., Ferreira T., Velez-Edwards D.R., Edwards T.L. (2019). Genome-wide association and epidemiological analyses reveal common genetic origins between uterine leiomyomata and endometriosis. Nat. Commun..

[50] Kho P.F., Mortlock S., Amant F., Annibali D., Ashton K., Attia J., Auer P.L., Beckmann M.W., Black A., Brinton L. (2021). Genetic analyses of gynecological disease identify genetic relationships between uterine fibroids and endometrial cancer, and a novel endometrial cancer genetic risk region at the WNT4 1p36.12 locus. Hum. Genet..

[51] Murji A., Bedaiwy M., Singh S.S., Bougie O., CAPTURE Registry Steering Committee (2020). Influence of Ethnicity on Clinical Presentation and Quality of Life in Women with Uterine Fibroids: Results from a Prospective Observational Registry. J. Obstet. Gynaecol. Can..

[52] Hellwege J.N., Jeff J.M., Wise L.A., Gallagher C.S., Wellons M., Hartmann K.E., Jones S.F., Torstenson E.S., Dickinson S., Ruiz-Narváez E.A. (2017). A multi-stage genome-wide association study of uterine fibroids in African Americans. Hum. Genet..

[53] Edwards T.L., Giri A., Hellwege J.N., Hartmann K.E., Stewart E.A., Jeff J.M., Bray M.J., Pendergrass S.A., Torstenson E.S., Keaton J.M. (2019). A Trans-Ethnic Genome-Wide Association Study of Uterine Fibroids. Front. Genet..

[54] Chan I., Wong T., Martinez-Mir A., Christiano A.M., McGrath J.A. (2005). Familial multiple cutaneous and uterine leiomyomas associated with papillary renal cell cancer. Clin. Exp. Dermatol..

[55] Tomlinson I.P., Alam N.A., Rowan A.J., Barclay E., Jaeger E.E., Kelsell D., Leigh I., Gorman P., Lamlum H., Rahman S. (2002). Germline mutations in FH predispose to dominantly inherited uterine fibroids, skin leiomyomata and papillary renal cell cancer. Nat. Genet..

[56] Menko F.H., Maher E.R., Schmidt L.S., Middelton L.A., Aittomäki K., Tomlinson I., Richard S., Linehan W.M. (2014). Hereditary leiomyomatosis and renal cell cancer (HLRCC). Renal cancer risk, surveillance and treatment. Fam. Cancer.

[57] Reyes C., Karamurzin Y., Frizzell N., Garg K., Nonaka D., Chen Y.-B., A Soslow R. (2014). Uterine smooth muscle tumors with features suggesting fumarate hydratase aberration: Detailed morphologic analysis and correlation with S-(2-succino)-cysteine immunohistochemistry. Mod. Pathol..

[58] Novel Mutations in FH and Expansion of the Spectrum of Phenotypes Expressed in Families with Hereditary Leiomyomatosis and Renal Cell Cancer|Journal of Medical Genetics. https://jmg.bmj.com/content/43/1/18.

[59] Lehtonen R., Kiuru M., Vanharanta S., Sjöberg J., Aaltonen L.-M., Aittomäki K., Arola J., Butzow R., Eng C., Husgafvel-Pursiainen K. (2004). Biallelic inactivation of fumarate hydratase (FH) occurs in nonsyndromic uterine leiomyomas but is rare in other tumors. Am. J. Pathol..

[60] (2011). Uterine Fibroids Gene Therapy: Targeted Adenovirus Vector (Ad-SSTR-RGD-TK/GCV) Provides Superior Inhibition of Human Leiomyoma Cells than Human Uterine Smooth Muscle Cells. Mol. Ther..

[61] Abdelaziz M., Sherif L., ElKhiary M., Nair S., Shalaby S., Mohamed S., Eziba N., El-Lakany M., Curiel D., Ismail N. (2016). Targeted Adenoviral Vector Demonstrates Enhanced Efficacy for In Vivo Gene Therapy of Uterine Leiomyoma. Reprod. Sci..

[62] Hassan M.H., Khatoon N., Curiel D.T., Hamada F.M., Arafa H.M., Al-Hendy A. (2008). Toward gene therapy of uterine fibroids: Targeting modified adenovirus to human leiomyoma cells. Human. Reprod..

[63] Egorova A., Shtykalova S., Maretina M., Freund S., Selutin A., Shved N., Selkov S., Kiselev A. (2023). Serum-Resistant Ternary DNA Polyplexes for Suicide Gene Therapy of Uterine Leiomyoma. Int. J. Mol. Sci..

[64] Duarte S., Carle G., Faneca H., de Lima M.C.P., Pierrefite-Carle V. (2012). Suicide gene therapy in cancer: Where do we stand now?. Cancer Lett..

[65] Hassan M., Zhang D., Salama S., Hamada F., Arafa H., Fouad H., Walker C., Al-Hendy A. (2009). Towards fibroid gene therapy: Adenovirus-mediated delivery of herpes simplex virus 1 thymidine kinase gene/ganciclovir shrinks uterine leiomyoma in the Eker rat model. Gynecol. Obstet. Investig..

[66] Włodarczyk M., Nowicka G., Ciebiera M., Ali M., Yang Q., Al-Hendy A. (2022). Epigenetic Regulation in Uterine Fibroids—The Role of Ten-Eleven Translocation Enzymes and Their Potential Therapeutic Application. Int. J. Mol. Sci..

[67] An J., González-Avalos E., Chawla A., Jeong M., López-Moyado I.F., Li W., Goodell M.A., Chavez L., Ko M., Rao A. (2015). Acute loss of TET function results in aggressive myeloid cancer in mice. Nat. Commun..

